# Validation of the Spanish version of the Irrational Procrastination Scale (IPS)

**DOI:** 10.1371/journal.pone.0190806

**Published:** 2018-01-05

**Authors:** Georgina Guilera, Maite Barrios, Eva Penelo, Christopher Morin, Piers Steel, Juana Gómez-Benito

**Affiliations:** 1 Departament de Metodologia de les Ciències del Comportament, Facultat de Psicologia, Universitat de Barcelona, Barcelona, Spain; 2 Institut de Neurociències (UBNeuro), Universitat de Barcelona, Barcelona, Spain; 3 Laboratori d’Estadística Aplicada, Departament de Psicobiologia i Metodologia de les Ciències de la Salut, Universitat Autònoma de Barcelona, Bellaterra, Spain; 4 Haskayne School of Business, University of Calgary, Calgary, Canada; Stellenbosch University, SOUTH AFRICA

## Abstract

The present study is centered in adapting and validating a Spanish version of the Irrational Procrastination Scale (IPS). The sample consists of 365 adults aged 18–77 years (*M* = 37.70, *SD* = 12.64). Participants were administered two measures of procrastination, the IPS and the Decisional Procrastination Questionnaire, as well as the Big Five Inventory, and the Satisfaction With Life Scale. First, the factor and replication analysis revealed that the internal structure of the scale is clearly one-dimensional, supporting the idea that IPS seems to measure general procrastination as a single trait. Second, the internal consistency is satisfactory as is the temporal stability of the IPS scores. Third, the correlations encountered between the IPS scores and other measures of procrastination, personality traits and satisfaction with life are all in the expected direction and magnitude. Finally, consistent with previous research, procrastination is related to age, with the youngest being the most procrastinating group. This study represents the first attempt in adapting and validating the IPS measure of procrastination into Spanish. Results suggest that the Spanish version of the IPS offers valid and reliable scores when applied to adult population.

## Introduction

Procrastination is defined as ‘‘to voluntarily delay an intended course of action despite expecting to be worse off for the delay” ([1], p. 66). Epidemiological studies establish that self-reported procrastination is present in at least 20% of the general adult population [2], and that chronic procrastination is common among English-speaking countries (i.e., Australia, United Kingdom, and United States) and Spanish-speaking countries (i.e., Peru, Spain, and Venezuela) where around 14% of adults self-identified as procrastinators [3]. In a Turkish sample, prevalence analysis revealed 15% of the adult population to be chronic procrastinators [4]. These studies used self-reported measures of procrastination, leaving out of their scope to what extent chronic procrastinators present a clinical condition that warrants intervention.

Despite the rise in recognition and importance, clarifying the exact nature and definition of procrastination and also its assessment has caused an intense debate in recent years [5]. In one of his pioneering studies, Ferrari [6] proposed differentiating between decisional (delays in making decisions), avoidant (delays related to fears of failure or success), and arousal (delays motivated by a "last-minute" thrill experience) procrastination. But current evidence does not support this tripartite model, especially regarding avoidant and arousal, instead indicating a new conceptualization of procrastination that postulates that procrastination is indeed an irrational delay [5].

When carrying out epidemiological studies, to have procrastination measurement instruments that provide valid and reliable measures is an essential issue. Examples of this kind of studies are those centered in determining population prevalence, as well as correlational studies focused on exploring the relationships between procrastination and other variables (e.g., socio-demographics, personality traits). Several measures have been developed in order to assess the occurrence and severity of self-reported procrastination, as well as to test the conceptual underpinnings of different theories of procrastination [7]. In a recent publication, Steel [5] identified five key procrastination scales and their revised versions: a) the Decisional Procrastination Questionnaire (DPQ) [8], consisting of 30 items measuring decisional procrastination; b) the General Procrastination Scale (GPS) [9], formed by 20 items that assess procrastination in general; c) the Procrastination Assessment Scale for Students (PASS) [10], 12 items measuring procrastination in curricular activities; d) the Adult Inventory of Procrastination (AIP) [11], 15 items that also measure general procrastination; and e) the Tuckman Procrastination Scale (TPS) [12], consisting of 16 items assessing procrastination tendencies. Psychometric properties of all these measures have been evaluated in their respective original versions, and they seem to function properly when applied to community adult populations. In Spanish, Díaz-Morales and colleagues [13] proposed translations for the GPS, the AIP and the DPQ, and tested its dimensional structure and internal consistency, concluding that the Spanish versions of the three procrastination scales provide effective and reliable measures. This was a first step in making available procrastination scales to researchers and professionals in general from Spanish-speaking countries. However, the results from Steel [5] suggest that a single latent variable is sufficient to explain the nature of procrastination (i.e., dysfunctional delay), and that self-report measures as those specified here (e.g., GPS or AIP) trying to differentiate between types of procrastination are unwarranted [7]. In this scenario, Steel [5] proposed two new measures of the “pure” procrastination or dysfunctional delay, including the Pure Procrastination Scale (PPS; i.e., derived by factor analyzing three existing instruments and retaining only those items with the highest loadings on the first factor), and the Irrational Procrastination Scale (IPS; i.e., a new measure of dysfunctional delay which proved functionally equivalent to the PPS). Hence, both PPS and IPS may become potential valuable instruments for determining the prevalence and severity of self-reported procrastination since they provide a single theoretical model and are easy to administer. The PPS has satisfactorily been translated and validated into seven European languages [7,14–16], and the IPS into Finnish, German, Indonesian, Italian, Norwegian, Polish, and Swedish [7,15–17], the last being used in a large international epidemiological study in eight English-speaking countries [18]; however, none of them has been adapted into the Spanish adult population. More efforts should be devoted to adapting them to other languages and cultures, including the Spanish-speaking context, allowing for worldwide cross-cultural comparisons of the phenomenon of procrastination.

The present study is centered in adapting and validating the Spanish version of the IPS, and the objectives are the following: 1) to present a Spanish version of the IPS, 2) to explore the dimensional structure of the scale, 3) to determine the internal consistency and stability of the scores, 4) to explore the relationships between the IPS and other self-report measures of delays in making decisions, personality traits and satisfaction with life, and 5) to examine the association of procrastination to demographic factors.

## Materials and methods

### Participants

The sample consisted of 365 adults aged 18–77 years (*M* = 37.70, *SD* = 12.64). Additional socio-demographic characteristics of the sample are shown in Table 1.

**Table 1:** Socio-demographic characteristics of the sample.

Variable	*n*	%	Variable	*n*	%
**Sex**			**Living arrangement**		
Male	147	40.3	Original family	76	20.8
Female	218	59.7	Own family	234	64.1
**Employment status**			Friends	23	6.3
Wage earner	202	55.3	Alone	30	8.2
Self-employed	46	12.6	Other	2	0.5
Non-paid work	14	3.8	**Educational level**		
Unemployed	51	14.0	Primary education not completed	1	0.3
Retired	17	4.7	Primary education	28	7.7
Housewife	3	0.8	Secondary education	102	27.9
Disability	2	0.5	Higher education	202	55.3
Other	30	8.2	Other	32	8.8

### Instruments

Participants were administered a battery of questionnaires (list available upon request). For the purposes of the present study, included were two measures of procrastination, the IPS [5] and the DPQ [8], as well as the Big Five Inventory (BFI) [19] and the Satisfaction With Life Scale (SWLS) [20].

The IPS [5] is formed by 9 items with a 5-point Likert scale (response categories range from 1 = “*very seldom*, *or not true of me*” to 5 = “*very often true*, *or true of me*”) assessing the degree of irrational delay causing procrastination. The scores of the English version have proven to be reliable, with a Cronbach’s alpha value of .91, and valid in terms of relations between the scale and other measures of procrastination (i.e., correlations ranged between .56 and .87) [5]. Several adaptations to other languages and cultures have been initiated (i.e., Finnish, German, Indonesian, Italian, Norwegian, Polish, and Swedish), and they have shown adequate psychometric properties in terms of dimensional structure and internal consistency [7,15–17].

The original version in English was translated into Spanish by two of the authors of the present study (MB and GG), and back-translated into English by a native English-speaking psychologist with excellent mastery of Spanish. The original and the back-translated English versions were compared, and in order to guarantee that the psychological meaning of both versions was captured, Dr. Piers Steel (author of the IPS) and Christopher Morin (PhD student under the supervision of Dr. Steel) were contacted to give their opinion in this sense. Some slight modifications were introduced in two items accordingly to their suggestions (personal communication, September 15, 2014). The final version of the Spanish IPS can be read in S1 Appendix.

The DPQ [8] (Spanish version by Díaz-Morales et al. [13]) is a scale that focuses on putting off decisions (e.g., “I don’t make decisions unless I really have to”) with 5 items in a 5-point Likert scale, ranging from 1 = “*not true for me*” to 5 = “*true for me*”. Both the original English and the adapted Spanish versions have shown good psychometric properties when applied to adult population. A previous study [5] has shown that the correlation between IPS and DPQ reaches a value of .69.

The BFI [19] (Spanish version by Benet-Martínez and John [21]) comprises 44 items in a 5-point Likert scale (from 1 = “*disagree strongly*” to 5 = “*agree strongly*”) assessing the big five personality dimensions: Extraversion (e.g., “I see myself as someone who is full of energy”), Agreeableness (e.g., “I see myself as someone who is generally trusting”), Conscientiousness (e.g., “I see myself as someone who can be somewhat careless”), Neuroticism (e.g., “I see myself as someone who is relaxed, handles stress well”), and Openness (e.g., “I see myself as someone who has an active imagination”). The BFI scores have proven to be valid in terms of internal structure and convergent validity, and reliable in terms of internal consistency (mean Cronbach’s alpha of .78) when applied to a sample of students. A comprehensive meta-analysis [1] has indicated that procrastination strongly correlates with Conscientiousness (*r* = −.62), weakly with Extraversion (*r* = −.12), Agreeableness (*r* = −.12), and Neuroticism (*r* = .24), while an absence of a relationship is shown with Openness (*r* = .03).

Finally, the SWLS [20] (Spanish version by Vázquez et al. [22]) is a short scale composed of five simple items measuring life satisfaction (e.g., “The conditions of my life are excellent”), with responses ranging from 1 = “*strongly disagree*” to 7 = “*strongly agree*”. Several studies have confirmed that the SWLS scores are reliable and valid (see the review of its psychometric properties by Pavot and Diener [23]). Steel [5] found that the IPS score was inversely related with the SWLS, obtaining a correlation coefficient of −.41.

### Procedure

For a self-report research with adults from a community-sample, the approval by Ethics Committee was not required at the time the study was conducted. Institutional review boards exempt researchers from approaching the committee for such kind of research. Adult participants were recruited using a convenience sampling (i.e., snowball approach), where a group of psychology undergraduates were offered to respond the questionnaires and also to invite people from their family and circle of friends to participate. They were informed about the nature of the research and the study’s objectives, and they gave written consent being made clear that participation was voluntary and that all data would remain confidential at all times. Sample recruitment extended from October 2014 through April 2015. After completing the questionnaires participants were given a report with their individual results. A subsample of 275 individuals agreed to participate in the retest phase, among which 122 participants (44.4% response rate; 32.8% males, 67.2% females) responded the questionnaires after between two weeks and approximately two months.

### Statistical analysis

The distribution of responses to IPS items was explored by obtaining the percentage of endorsement of each response category, skewness and kurtosis, and of the IPS total score was assessed in terms of mean, standard deviation, observed range, and levels of skewness and kurtosis. To examine the dimensional structure of the scale and its replicability, the sample was randomly split into two subsamples (Subsample 1: *n* = 182, Subsample 2: *n* = 183). Two independent exploratory factor analyses were performed using the preferred maximum likelihood factor extraction method [24]. For determining the number of factors to retain, the Minimum Average Partial (MAP) procedure [25] was utilized. In order to assess replicability of factor loadings over subsamples, the magnitude of factor loadings were compared obtaining the squared differences between corresponding items within each factor [26]. Internal consistency was assessed obtaining the Cronbach’s alpha coefficient, and its 95% confidence interval (95% CI) was also calculated. To explore the test-retest reliability the intraclass correlation coefficient (i.e., single measures of absolute agreement) was computed relating scores from both administration times. The IPS was also related with the DPQ, BFI and SWLS scores obtaining the Pearson correlation coefficient. Coefficients both uncorrected and corrected for attenuation due to unreliability were reported, applying in the last case the following formula: $r_{x^{\prime }y^{\prime }}=r_{xy}\sqrt{r_{xx}r_{yy}}$ [27], and were interpreted following Cohen’s criteria [28]. In order to confirm the results reported by Beutel and colleagues [29] in terms of differences in procrastination between participants grouped by sex and age, the variable age was categorized in two groups (i.e., 18–29 [the only group that showed differences with all the other age groups in Beutel’s study], and 30–77). A two-factor ANOVA was conducted using the IPS score as dependent, and sex and age group as independent variables. Effect sizes *d* were interpreted according to Cohen’s criteria [28]. The *psych* package for R [30] was used for the implementation of the MAP procedure, and SPSS version 21 for the remaining analyses.

## Results

### Distribution of scores

The distribution of responses in each IPS item and levels of skewness and kurtosis are shown in Table 2. Item endorsements covered all the spectrum of response categories. Responses in item IPS1 were highly concentrated in the first three response categories (almost 90%), similarly to item IPS9 in the last three categories (94%), but the remaining items had a good coverage among the most categories. Skewness and kurtosis values were within the range to consider the IPS items’ responses as not deviated from normality.

**Table 2:** Item endorsements in each response category and corresponding skewness and kurtosis values.

	Response category n (%)						
Item	1	2	3	4	5	Skewness	Kurtosis
**IPS1**	109 (29.9)	143 (39.2)	77 (21.1)	29 (7.9)	7 (1.9)	.704	−.002
**IPS2 (R)**	48 (13.2)	168 (46.0)	106 (29.0)	36 (9.9)	7 (1.9)	.516	.091
**IPS3**	57 (15.6)	94 (25.8)	133 (36.4)	46 (12.6)	35 (9.6)	.267	−.545
**IPS4**	40 (11.0)	131 (35.9)	134 (36.7)	50 (13.7)	10 (2.7)	.255	−.242
**IPS5**	16 (4.4)	108 (29.6)	137 (37.5)	76 (20.8)	28 (7.7)	.246	−.468
**IPS6 (R)**	31 (8.5)	127 (34.8)	135 (37.0)	57 (15.6)	15 (4.1)	.290	−.250
**IPS7**	52 (14.2)	166 (45.5)	93 (25.5)	45 (12.3)	9 (2.5)	.544	−.113
**IPS8**	62 (17.0)	136 (37.3)	115 (31.5)	43 (11.8)	9 (2.5)	.351	−.326
**IPS9 (R)**	58 (15.9)	210 (57.5)	75 (20.5)	19 (5.2)	3 (0.8)	.739	.983

IPS: Irrational Procrastination Scale

1: *Very seldom*, *or not true of me*; 2: *Seldom true of me*; 3: *Sometimes true of me*; 4: *Often true of me*; 5: *Very often true*, *or true of me*

For reversed items (R) 1: *Very often true*, *or true of me*; 2: *Often true of me*; 3: *Sometimes true of me*; 4: *Seldom true of me*; 5: *Very seldom*, *or not true of me*

The mean total score of the IPS was 22.67 (*SD* = 6.55), with an observed range between 9 and 45, a skewness value of *S* = 0.584 and a kurtosis of *K* = 0.224.

### Dimensional structure

Prior to conducting the exploratory factor analyses, the Kaiser-Meyer-Olkin (KMO) measure of sampling adequacy and the Bartlett’s test of sphericity were calculated. For Subsample 1, KMO = .902, Barlett’s test *χ*^*2*^(36) = 873.61, *p* < .001; for Subsample 2, KMO = .919, Barlett’s test *χ*^*2*^(36) = 929.03, *p* < .001. These results support the pertinence of conducting both factor analyses. The factor analysis of Subsample 1 revealed a single factor with an eigenvalue of 4.619 that accounted for 51.32% of the observed variance; for Subsample 2 one factor was also extracted with an eigenvalue of 4.827 and 53.64% of the total variance explained. In this regard, the MAP procedure indicated the appropriateness of extracting a single factor in both datasets. Item-total correlations ranged from .499–.802 (Subsample 1) and .504–.839 (Subsample 2). Table 3 presents the communalities and factor loadings for the two samples, as well as the squared differences between the loadings. The factor loadings for Subsample 1 ranged between .511 (item IPS9) and .867 (item IPS7); for Subsample 2 they ranged between .517 (item IPS3) and .898 (item IPS7). Additionally, as can be seen from the squared differences between the factor loadings, all IPS items had values below .04 criterion value.

**Table 3:** Communalities and factor loadings in each subsample, and the squared differences between loadings.

	Subsample 1 (*n* = 182)		Subsample 2 (*n* = 183)			
Item	Communalities	Loadings	Communalities	Loadings	Squared differences between loadings
**IPS1**	.429	.655	.493	.702	.002	
**IPS2 (R)**	.380	.617	.425	.652	.001	
**IPS3**	.335	.579	.267	.517	.004	
**IPS4**	.584	.764	.678	.823	.003	
**IPS5**	.634	.796	.497	.705	.008	
**IPS6 (R)**	.503	.710	.533	.730	.000	
**IPS7**	.752	.867	.806	.898	.001	
**IPS8**	.740	.860	.701	.837	.001	
**IPS9 (R)**	.262	.511	.428	.654	.020	

Item scores were reversed (R) before running the exploratory factor analysis

Thus, both datasets supported a one factor solution of the IPS items, reflecting a single latent variable. The comparisons of individual factor loadings indicated that the IPS items load similarly over both subsamples, so factor loadings are highly replicable.

### Internal consistency and Temporal stability

The Cronbach’s alpha coefficient reached a value of .90 (95% CI: .88–.92), indicating an excellent internal consistency of the IPS scores. Additionally, each item significantly correlated with the IPS corrected total score, ranging from .530 to .819, and the alpha value decreased or remained equal (from .88 to .90) with the removal of individual items, together supporting the good internal consistency of the scale scores. ICC correlation coefficient for the scale administered at two time points was .84, a value that denotes a high stability of IPS total score.

### Relations with other measures

The IPS was correlated with another measure of procrastination (DPQ), the five personality factors of the BFI, and a satisfaction with life scale (SWLS). Table 4 shows the reliability estimates for all of the included scales, and also the correlation coefficients between these instruments and the IPS, both uncorrected and corrected for attenuation due to unreliability of measures. As expected, IPS scores strongly correlated with the DPQ and the Conscientiousness factor of the BFI (uncorrected: .69 and −.72, respectively; corrected: .62 and −.62, respectively), and moderately related with the SWLS (−.36 and −.32, respectively for uncorrected and corrected coefficients). Correlations of the IPS with the BFI factors Extraversion, Agreeableness, and Neuroticism were all low, and almost null with the Openness factor.

**Table 4:** Internal consistency and correlations between the IPS and other measures of procrastination, personality traits and satisfaction with life.

Measure	Cronbach’s alpha	Uncorrected r_xy_	Corrected for unreliability r_x’y’_
**DPQ**	.90	.69	.62
**BFI-Extraversion**	.84	−.24	−.21
**BFI-Agreeableness**	.68	−.22	−.17
**BFI-Conscientiousness**	.82	−.72	−.62
**BFI-Neuroticism**	.85	.24	.21
**BFI-Openness**	.81	−.06	−.05
**SWLS**	.86	−.36	−.32

DPQ: Decisional Procrastination Questionnaire; BFI: Big Five Inventory; SWLS: Satisfaction With Life Scale

Males scored higher than females [*M* = 24.03, *SD* = 6.25 *vs*. *M* = 21.75, *SD* = 6.60, respectively; *F*(1,361) = 3.96, *p* < .05] in IPS scores, obtaining a low to moderate effect size (*d* = 0.35). Furthermore, a statistically significant effect of age was observed [*F*(1,361) = 15.60, *p* < .01], the scores being higher in the youngest group (*M* = 25.51, *SD* = 6.55) than in the oldest group (*M* = 21.69, *SD* = 6.27) with a medium effect size (*d* = 0.60). Even though the interaction term (sex × age) was not statistically significant [*F*(1,361) = 0.63, *p* = .43], differences between males and females tended to be more prominent in the youngest and most procrastinating group, with an effect size of *d* = 0.35 compared to that found in the oldest group (*d* = 0.16).

## Discussion

This study represents the first attempt in adapting and validating the IPS measure of procrastination into Spanish. Results suggest that the Spanish version of the IPS offers valid and reliable scores when applied to an adult population.

First, factor analysis reveals that the internal structure of the scale is clearly one-dimensional, supporting the idea of Steel [5] and Svartdal and colleagues [15,16] that IPS seems to measure general procrastination as a single trait. The studies from Prayitno et al. [17] and Rozental et al. [7] encountered a two-dimensional structure, but they argued that the second factor may be an artifact of the instrument (e.g., due to the presence of reverse items) and they both postulate that a one-dimensional structure is more plausible. Actually, characteristics of samples used in these studies are far from those analyzed with the English and Spanish versions, i.e., Prayitno et al. [17] administered the IPS to a sample of students, and Rozental et al. [7] used a clinical sample. These differences may explain the disparity of results in terms of dimensional structure.

Second, the internal consistency is satisfactory as is the temporal stability of the IPS scores. In relation to internal consistency, our results again are more similar to those encountered by Steel [5] (i.e., .91) and Svartdal and colleagues [16] (i.e., range over countries: .85−.93), than to those lower scores encountered with the Indonesian and Swedish versions (i.e., .79 and .76, respectively) [7,17]. The ICC found in the present study is practically equal to the .83 reported by Rozental and colleagues [7].

Third, the correlations encountered between the IPS scores and other measures of procrastination, personality traits and satisfaction with life are all in the expected direction and magnitude. The relationship between the two measures of procrastination (i.e., IPS and DPQ) is strong, reaching a value of .69 which is at the same level as that reported by Steel [5], providing evidence of convergent validity. The correlations observed with the big five personality traits are also in line with those reported in two separate meta-analyses by Van Eerde [31] and Steel [1], indicating that procrastination only presents a relevant relationship with the factor Conscientiousness. Additionally, Steel [5] and Svartdal [15] found that the IPS score was inversely related with the SWLS, obtaining a correlation coefficient of −.41 and −.35 respectively, values that are very close to the one reported in the present study. This later result is also in agreement with recent studies [14,29] which suggested an inverse relationship between procrastination and life satisfaction.

Finally, our results are also in accord with previous research [16,18,29], showing that the youngest group (i.e., 18–29) was the most procrastinating, being the level of procrastination represented by the IPS scores, and also in this group males tended to procrastinate slightly more than females.

The current study has several limitations that should be noted. First, participants were recruited using a convenience sampling, which may have influenced the results obtained, since only those more interested may have participated to a greater extend. In addition, the sample was imbalanced in terms of sex (approximately 40% men *vs*. 60% women). Second, convergent validity of the IPS scores was assessed by means of the DPQ, an instrument that only encompasses the decision making component of procrastination. Third, all instruments were self-report measures. Since a previous study has demonstrated a lack of convergence between self-assessed and observed procrastination [32], results of the present study can only be considered valid for self-reported procrastination. In future work it will be necessary to assess the generalization of the results, evaluating the IPS usefulness and psychometric properties in other samples and contexts. Moreover, in order to accumulate validity evidence of the scale scores, further validity studies should incorporate other procrastination measures, such as the PPS, and it would also be relevant to explore the relationship between the self-report IPS and behavioral measures of procrastination [33], or the link between procrastination and other related variables, such as the consideration of future consequences [34].

Despite these limitations, the present study provides preliminary validity and reliability evidence for the Spanish version of the IPS scores. In this sense, the IPS offers a simple and inexpensive tool that could easily permit mass testing in large population based-epidemiological studies [18], also allowing for cross-cultural comparisons [16].

## Conclusions

The Spanish adaptation of the IPS proposed in the present study shows reliable and valid scores for assessing procrastination in adult population. Taking into account that Spanish is spoken by over 470 million people worldwide, the Spanish version of the IPS can be effectively used in several assessment settings involving a wide range of Spanish speaking populations. Further evidence should be collected in order to explore its functioning in other settings such as clinical contexts.

## Supporting information

S1 Appendix The Spanish version of the Irrational Procrastination Scale. (DOCX)
