# Supplementary material for: Validation of the Spanish version of the Irrational Procrastination Scale (IPS)
Source: PLoS One. 2018 Jan 5;13(1):e0190806. doi: 10.1371/journal.pone.0190806 (PMC5755900; doi:10.1371/journal.pone.0190806)
Supplement: S1 Appendix — (DOCX) [file pone.0190806.s001.docx]

**S1 Appendix. The Spanish version of the Irrational Procrastination Scale**

A continuación encontrará una serie de afirmaciones. Indique hasta qué punto está de acuerdo o en desacuerdo con cada afirmación seleccionando la respuesta que mejor describe su forma de ser y hacer. Por favor, responda siguiendo la siguiente escala:

1= No me describe en absoluto / 2= No es usual en mí / 3= A veces sí, a veces no / 4= Es usual en mí / 5= Me describe totalmente

1. Pospongo tanto las cosas que mi bienestar o eficiencia se ven afectados innecesariamente
2. Si hay algo que debo hacer, lo hago antes que atender a tareas menos importantes (R)
3. Si algunas cosas las hubiera hecho antes, mi vida sería mejor
4. Cuando debería estar haciendo una cosa, me pongo a hacer otra
5. Al final del día, sé que podría haberme distribuido mejor el tiempo
6. Me organizo el tiempo adecuadamente (R)
7. Retraso las tareas más de lo que sería razonable
8. Dejo para mañana lo que tendría que hacer hoy
9. Hago las cosas cuando creo que hay que hacerlas (R)

*Note.* Items designated with an (R) are reverse scored.
